# Diversity of European habitat types is correlated with geography more than climate and human pressure

**DOI:** 10.1002/ece3.8409

**Published:** 2021-12-07

**Authors:** Marco Cervellini, Michele Di Musciano, Piero Zannini, Simone Fattorini, Borja Jiménez‐Alfaro, Emiliano Agrillo, Fabio Attorre, Pierangela Angelini, Carl Beierkuhnlein, Laura Casella, Richard Field, Jan‐Christopher Fischer, Piero Genovesi, Samuel Hoffmann, Severin D. H. Irl, Juri Nascimbene, Duccio Rocchini, Manuel Steinbauer, Ole R. Vetaas, Alessandro Chiarucci

**Affiliations:** ^1^ BIOME Lab, Department of Biological, Geological and Environmental Sciences, Alma Mater Studiorum University of Bologna Bologna Italy; ^2^ Department of Life, Health and Environmental Sciences University of L’Aquila L’Aquila Italy; ^3^ Research Unit of Biodiversity (CSIC/UO/PA) University of Oviedo Mieres Spain; ^4^ Institute for Environmental Protection and Research (ISPRA) Rome Italy; ^5^ Department of Environmental Biology Sapienza University of Rome Roma Italy; ^6^ Biogeography, Bayreuth Center of Ecology and Environmental Research (BayCEER), Geographical Institute Bayreuth (GIB) University of Bayreuth Bayreuth Germany; ^7^ School of Geography University of Nottingham Nottingham UK; ^8^ School of Earth Sciences University of Bristol Bristol UK; ^9^ Biogeography and Biodiversity Lab, Institute of Physical Geography Goethe‐University Frankfurt Germany; ^10^ Department of Spatial Sciences, Faculty of Environmental Sciences Czech University of Life Sciences Prague Praha Czech Republic; ^11^ Sport Ecology, Bayreuth Center of Ecology and Environmental Research (BayCEER) & Department of Sport Science University of Bayreuth Bayreuth Germany; ^12^ Department of Geography University of Bergen Bergen Norway

**Keywords:** anthropogenic impact, biodiversity conservation, environmental predictors, European habitat directive, habitat richness, terrain ruggedness index

## Abstract

Habitat richness, that is, the diversity of ecosystem types, is a complex, spatially explicit aspect of biodiversity, which is affected by bioclimatic, geographic, and anthropogenic variables. The distribution of habitat types is a key component for understanding broad‐scale biodiversity and for developing conservation strategies. We used data on the distribution of European Union (EU) habitats to answer the following questions: (i) how do bioclimatic, geographic, and anthropogenic variables affect habitat richness? (ii) Which of those factors is the most important? (iii) How do interactions among these variables influence habitat richness and which combinations produce the strongest interactions? The distribution maps of 222 terrestrial habitat types as defined by the Natura 2000 network were used to calculate habitat richness for the 10 km × 10 km EU grid map. We then investigated how environmental variables affect habitat richness, using generalized linear models, generalized additive models, and boosted regression trees. The main factors associated with habitat richness were geographic variables, with negative relationships observed for both latitude and longitude, and a positive relationship for terrain ruggedness. Bioclimatic variables played a secondary role, with habitat richness increasing slightly with annual mean temperature and overall annual precipitation. We also found an interaction between anthropogenic variables, with the combination of increased landscape fragmentation and increased population density strongly decreasing habitat richness. This is the first attempt to disentangle spatial patterns of habitat richness at the continental scale, as a key tool for protecting biodiversity. The number of European habitats is related to geography more than climate and human pressure, reflecting a major component of biogeographical patterns similar to the drivers observed at the species level. The interaction between anthropogenic variables highlights the need for coordinated, continental‐scale management plans for biodiversity conservation.

## Graphical abstract

We modeled EU habitat richness at continental scale as a function of geographic, climatic, and anthropogenic variables. We found geographical variables were by far the most strongly correlated with habitat richness, followed by climate. However, anthropogenic variables gained importance when considering their interactions, with important implications for conservation planning.

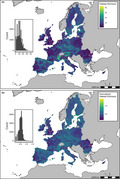

## 1 INTRODUCTION

The need to preserve dynamic ecosystems under changing climates and increasing anthropogenic pressure challenge traditional conservation approaches that are based on the current distribution of species. An application‐oriented way forward may lie in protecting those landscape elements that support the coexistence of many species. Indeed, the habitat (or ecosystem) approach for conservation has been recently highlighted by the IUCN as a necessary step for conservation. Under this view, habitat diversity is a complex, spatially explicit measure of biodiversity (Bunce et al., 2013), which has proven to be a prominent driver for species diversity of a variety of taxa at the landscape scale (Alsterberg et al., 2017; Dianzinga et al., 2020; Gibb et al., 2020; Keppel et al., 2016; Kerr & Packer, 1997).

According to the EU Habitats Directive (Council Directive 92/43/EEC), the term “habitat” refers to an environmental unit defined by specific abiotic and biotic factors. Although alternative definitions exist (Davies et al., 2004; Drakou et al., 2011; Hall et al., 1997; Kearney, 2006; Mitchell, 2005; Yapp, 1922), this formulation provides a pragmatic operational tool for characterizing landscape elements of conservation priority.

Term “habitat” in the context of the EU Habitats Directive has a particular meaning, which deviates from the autecological species‐related concept “habitat” in ecology. As an “environmental unit” that includes species assemblages and site conditions, the term “habitat” used in this context is closer to the concept of ecosystems (sensu Keith et al., 2013), even if some units are rather defined by mere plant communities (reflected in phytosociological terminology) (e.g., 6190 “Rupicolous pannonic grasslands (*Stipo*‐*Festucetalia pallentis*)”) and others are classified based on abiotic site conditions (e.g., 8240 “Limestone pavements,” 8320 “Fields of lava and natural excavations”), places (e.g., 8310 “Caves not open to the public”), or geographical units (e.g., 1150 “Coastal lagoons,” 1620 “Boreal Baltic islets and small islands”). Some units are characterized by vegetation structures (e.g., 5400 “Phrygana”) while others by typical species (e.g., 6160 “Oro‐Iberian *Festuca indigesta* grasslands”).

For the operational meaning of habitats, fluxes of energy and matter, and the processes that are forming an ecosystem, are not considered, even if these might be important (e.g., carbon sequestration, evapotranspiration). The concept of habitat is thus more focused on ecological compartments with a main emphasis on vegetation. Being aware of this inconsistency, using habitat types as indicated by EU Habitats Directive, provides a standardized and legally established tool for monitoring and assessment complex units, which is crucial for nature conservation.

Habitat diversity can be measured as the number of different habitats in a given area (Hortal et al., 2009; Triantis et al., 2006)—herein referred to as “habitat richness.” Habitat richness can be monitored in situ or by remote sensing techniques (Jung et al., 2020; Radeloff et al., 2019; Tuanmu & Jetz, 2015). Moreover, the accessibility of habitat distribution data is steadily growing, often provided in the form of maps, which may sometimes be proxies for—and the only available information on—the distribution of specific groups (e.g., plants and invertebrates).

Habitat richness reflects environmental conditions and can be used as an explanatory variable for modeling the distributions and abundances of species or communities (Heidrich et al., 2020; Leclère et al., 2020). Many studies have focused on the factors regulating the spatial variation in species richness (Brown & Lomolino, 2005; Field et al., 2009; Gaston, 2000; Howard et al., 2020; Quintero & Jetz, 2018; Rosenzweig, 1995). Species richness is typically correlated with variables such as climate (Gao & Liu, 2018; Thuiller et al., 2005), latitude (Gaston, 2000, 2007; Hillebrand, 2004), topographic heterogeneity (Hortal et al., 2009), and anthropogenic pressure (Liu et al., 2018; Malavasi et al., 2016). Contextually, the habitat amount hypothesis (Fahrig, 2013) predicts that species richness in equal‐sized sample sites should increase with the total amount of habitat. Similarly, the number of species inhabiting a region can be explained by the number of vegetation types as a surrogate of habitat diversity (Jiménez‐Alfaro et al., 2016). However, the relative importance of habitat amount and its spatial configuration (e.g., fragmentation, connectivity, or perimeter/area ratio) on biodiversity patterns is still subject of debate (Fahrig, 2021; Saura, 2021a, 2021b).

Despite the importance of habitat richness and the large amount of spatial data available for many terrestrial habitats, there is a knowledge gap on the mechanisms that determine the spatial patterns of habitat richness, especially at continental scales. To our knowledge, no research has been done on the underlying factors associated with habitat richness at continental level. In Europe, biodiversity conservation policy focuses on habitat protection (e.g., Council Directive 92/43/EEC), and achieving measurable improvements of the conservation status of Natura 2000 habitats is one of the main targets of the 2030 Biodiversity Strategy. As mandatory biodiversity monitoring, every EU member country is obliged to provide on a regular basis (every six years—art. 17 Habitat Directive) habitat distribution maps on a 10 km × 10 km grid map. Thus, disentangling the role of environmental factors determining habitat richness is a key challenge both for basic understanding of biodiversity distribution and for guiding conservation strategies (Mücher et al., 2009; Zhang et al., 2020).

To investigate how habitat richness is correlated with environmental factors across the EU, we used habitat distribution data from the third report of the EU Habitats Directive (EEA, 2020a). Specifically, we seek to answer the following questions: (i) how do bioclimatic, geographic, and anthropogenic variables affect habitat richness? (ii) Which factor is the most important? (iii) How do interactions among these variables influence habitat richness and which combinations produce the strongest interactions?

## 2 MATERIALS AND METHODS

### 2.1 Habitat richness

We used habitat distribution maps of 222 terrestrial habitats of community interest from the EU 2007–2012 reporting period, obtained from the European Environment Agency (EEA, 2020a). Distribution of the protected habitat type in Europe is based on the standard grid (10 km × 10 km) provided by EEA for habitat monitoring (EEA, 2013), and we assume it is representative of the whole ecosystem diversity. From a total of 231 habitat types, 9 marine habitats were excluded according to the appendix 2 of the “Lists of existing marine Habitat types and Species for different Member States” (European Commission, 2021). We calculated EU habitat richness (thereafter “habitat richness”) by summing up the number of habitat type reported in each cell of the above‐mentioned map provided by the EEA. All the EU countries that contributed to the third report were included, except Greece due to lack of habitat reporting there.

Despite being composed of equal‐area cells (i.e., 10 km × 10 km), the EEA grid has many cells located in coastal areas, often reducing the terrestrial surface within the cell. It has been widely reported that both species richness and habitat richness increase as a function of area (Lomolino, 2000; Rosenzweig, 1995; Triantis et al., 2012). Because of the effect of area on habitat richness, we applied a modified log–log power function (Arrhenius, 1921; Triantis et al., 2012) to normalize habitat richness values based on within‐cell land area. This involved adding 1 to habitat richness in order to have a normalized value of 0 when no habitat was present (instead of having minus infinite) and the absolute value of the denominator in order not to have inconsistent values when area was between 0 and 1 km^2^.

(1) $$\text{Normalized Habitat Richness}\;\left(\text{NHR}\right)=\frac{\log10\left(\text{HR}+1\right)}{\left|\log10\left(\text{area}\right)\right|}$$

### 2.2 Relations with species richness

Species richness data were obtained from the distributions of species reported in the Annexes of the Birds Directive (Council Directive 2009/147/EC; Annex I to V) and the Habitats Directive (Council Directive 92/43/EEC; Annex II, IV, and V) reported by EU member states for 2007–2012 (EEA, 2020a, 2020b). Further details can be found in Hoffmann et al. (2018). To test the assumption that habitat richness is a proxy of biodiversity conservation status, we calculated Pearson's *r* correlation coefficient (Pearson, 1931) to assess the correlation between habitat richness and species richness.

### 2.3 Environmental variables

We investigated the relationship between habitat richness and three groups of environmental variables: bioclimatic, geographic, and anthropogenic. Multicollinearity among variables was assessed through Pearson's *r*. Within variable pairs holding Pearson's *r* > .7 (see Figure S1), the one judged to have less ecological importance was discarded from model building (Dormann et al., 2013; Elith et al., 2006).

We extracted the 19 bioclimatic variables from WorldClim (Fick & Hijmans, 2017) at 10 km × 10 km resolution. Through variable selection (see Brandt et al., 2017), we ensured balanced representation of temperature and precipitation (both mean and seasonal variation—see Table 1 for a summary of the explanatory variables).

**TABLE 1:** Summary of explanatory and response variables. In bold variables selected for model building

Group	Acronym	Description	Type	Data source
Bioclimatic	**BIO_1**	Mean Annual Temperature	Numeric	Fick and Hijmans (2017)
Bioclimatic	BIO_4	Temperature Seasonality	Numeric	
Bioclimatic	**BIO_7**	Temperature Annual Range	Numeric	
Bioclimatic	**BIO_12**	Annual Precipitation	Numeric	
Bioclimatic	**BIO_15**	Precipitation Seasonality	Numeric	
Bioclimatic	BIO_17	Precipitation of Driest Quarter	Numeric	
Anthropogenic	**FRAG_IND**	Landscape Fragmentation Indicator	Raster	EEA (2011) and Jaeger (2000)
Anthropogenic	STREET_LENGTH	Total street length	Shapefile	Meijer et al. (2018)
Anthropogenic	STREET_DENSITY	Total street length divided by the area of a 10 km x 10 km cell	Shapefile	Meijer et al. (2018)
Anthropogenic	**POP_DENS**	Population density	Raster	EEA (2009) and Gallego (2010)
Geographic	**TRI**	Terrain Ruggedness Index	Derived from DEM	EU‐DEM (2021)
Geographic	**NORTH**	Northing		/
Geographic	**EAST**	Easting		/
Response	HAB_RICH	Habitat richness	Count	/
Response	**NORM_HAB_RICH**	Habitat richness normalized	Variables	/

Geographic variables, such as latitude and longitude, strongly affect species and habitat richness at different spatial scales (Drakou et al., 2011). Moreover, northing and easting (i.e., latitude and longitude, respectively, as geographic Cartesian coordinates) may be used to account for spatial autocorrelation. For these reasons, we included these two factors as explanatory variables in all the models. In order to capture geographic heterogeneity (Dufour et al., 2006), we included the terrain ruggedness index (TRI—Riley et al., 1999). TRI is defined as the mean of the absolute differences in elevation changes (Riley et al., 1999), and it is highly positively related to elevation (Amatulli et al., 2018). To obtain the Europe‐wide TRI, we used a freely available, 20‐m resolution digital elevation model (DEM) for the EU (EU‐DEM, 2021). We resampled the DEM to 200 m × 200 m pixel size using the arithmetic mean for aggregation. Then, for each 10 km × 10 km grid cell, we summed all the 2500 values obtained for TRI to obtain the topographic heterogeneity within each cell.

Anthropogenic factors and their interaction with bioclimatic and geographic variables strongly affect animal and plant biodiversity at global terrestrial scale (Mantyka‐Pringle et al., 2012, 2013). Three ‘anthropogenic’ variables were considered: landscape fragmentation index (LFI), population density, and total street length and street density (Table 1). Landscape fragmentation was extracted from EEA (EEA, 2011), and it was calculated based on the Effective Mesh Density, which is a measure of the degree to which movement between different parts of the landscape is interrupted by fragmentation geometry (FG) (Jaeger, 2000). The more FGs fragment the landscape, the higher the effective mesh density and hence the fragmentation (EEA, 2011; Jaeger, 2000; Moser et al., 2005; Schmiedel & Culmsee, 2016). Population density was also extracted from EEA (EEA, 2009). Both landscape fragmentation and population density were upscaled from 1 km^2^ to 100 km^2^ resolution, aggregating cells by median values due to their skewed distributions. Data on street length were extracted from the “Global biodiversity model for policy support” (GLOBIO—Meijer et al., 2018). Total street length was calculated by combining the length of all street types within each cell (highways, primary, secondary, tertiary, and local roads). Street density was calculated dividing the total street length by the area of each cell.

### 2.4 Data analysis

To test the significance of habitat richness as proxy of biodiversity, we first analyzed the relation between habitat richness and the richness of the species listed in the Annex species of the Birds and Habitats Directives, as reported by the single countries, by using Pearson correlation coefficient.

We then used generalized linear models (GLMs), generalized additive models (GAMs), and generalized boosted models (GBMs) to investigate how habitat richness responds to the selected climatic, geographic, and anthropogenic variables at the EU scale.

To identify the interactions to be included in the models, we ran all the possible combinations of variable pairs using the *glmulti* function from the *glmulti* package (Calcagno & de Mazancourt, 2010). To assess the best settings for the GBM models, all the possible combinations of three different numbers of trees (10,000, 15,000, and 20,000), four interaction depths (3, 5, 7, and 9), three shrinkages (0.01, 0.1, and 0.5) and three bag fractions (0.65, 0.8, and 1) were employed. We selected the combination with the lowest root mean square error (RMSE).

We run autocovariate models to filter out spatial dependence (Harisena, 2021). These models are usually based on autocovariate estimation directly on the response variable. Crase et al. (2012) developed a related procedure, in which autocovariates are quantified from the model residuals, rather than the raw data. This leads to an autocovariate that captures only the variance not explained by explanatory variables (Fletcher & Fortin, 2018). Moreover, models including autocovariates typically provide unbiased estimates of fixed effects, as demonstrated by Bardos et al. (2015). To fit autocovariate models, we calculated autocovariates on the model residuals and then we used these covariates in a new model. These autocovariates were calculated using the *autocov_dist* function in the *spdep* package (Bivand & Wong, 2018).

Variable importance for GLMs and GAMs was calculated excluding explanatory variables one by one from the models. Then, the contribution of each variable was assessed using the difference in deviance (*D* ^2^) between the model with and without that variable (Márcia Barbosa et al., 2013).

To produce response curves, we used the modified inflated response curve (Zurell et al., 2012) for abundance models. Interaction curves were produced by setting the explanatory variables to their mean values.

In addition, we calculate Pearson's *r* correlation for each candidate explanatory variable (1st and 2nd order polynomials) in order to show the bivariate relationship between normalized habitat richness and the entire set of environmental variables (Figure S3).

Data processing was performed with R 3.6.3 (R Core Team, 2020), using the following packages: *glmmulit* (Calcagno & de Mazancourt, 2010), *gbm3* (Hickey, 2016), *ggplot2* (Wickham, 2016), *ggeffect* (Lüdecke, 2018), *plot3D* (Soetaert, 2019), *tidyverse* (Wickham et al., 2019), *dplyr* (Wickham et al., 2020), *lhs* (Carnell, 2020), patchwork (Pedersen, 2020), *mgcv* (Wood, 2020), and *GGally* (Schloerke et al., 2021).

## 3 RESULTS

**FIGURE 1:**
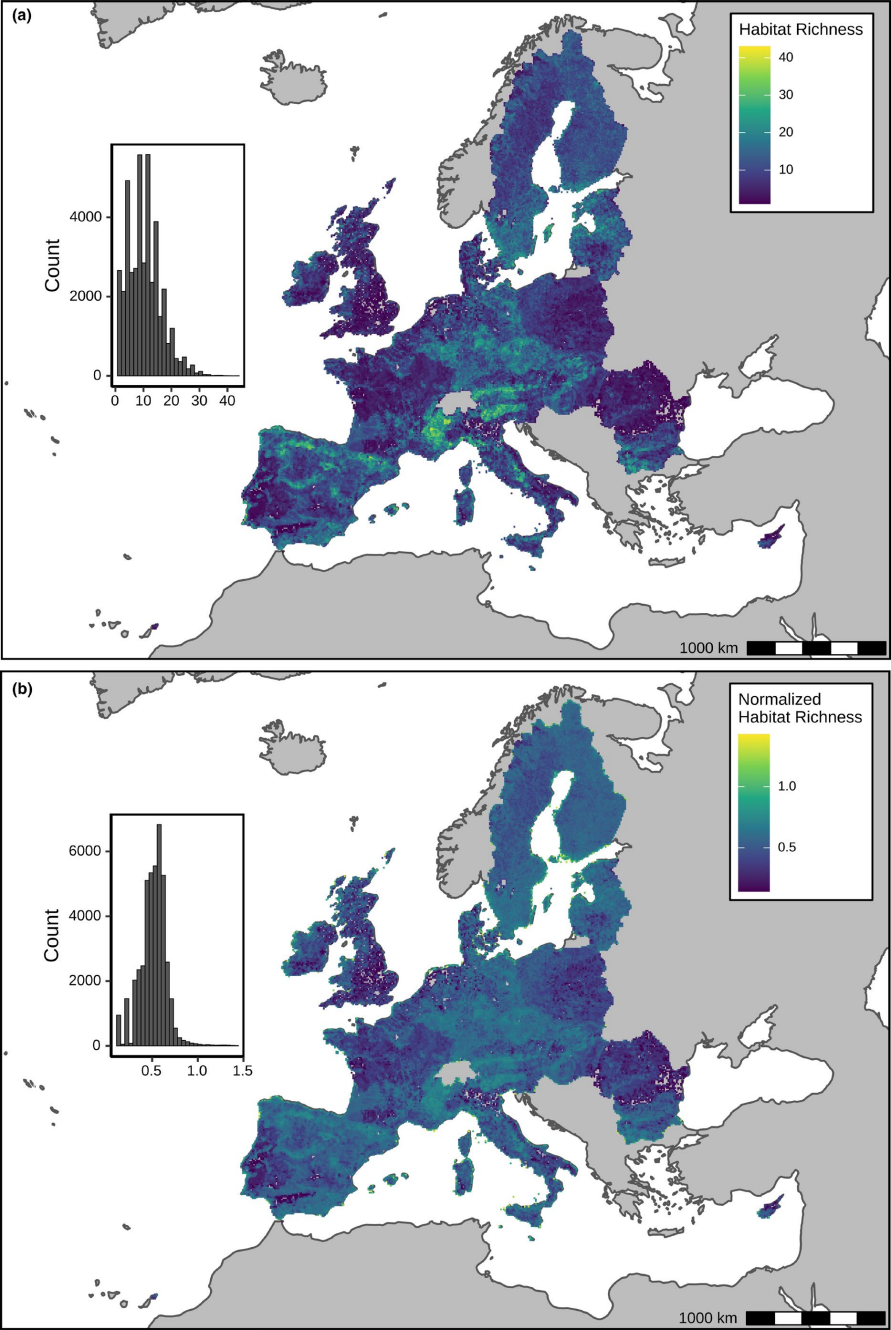
Maps of habitat richness obtained by overlapping habitat distribution maps with the standard grid of 10 km × 10 km cells provided by the European Environmental Agency (EEA) for habitat monitoring. Greece was excluded due to the lack of habitat reporting (lack of habitat reporting delivery of Article 17 data in 2013). Panel a shows the habitat richness. Panel b shows normalized habitat richness

Among the member countries of the European Union (EU, excluding Greece and including the former member United Kingdom), habitat richness (or habitat type richness; see Methods) per 10 km × 10 km cell (*N* = 43,240 cells in total) ranged from 0 to 43 (Figure 1a). While normalized habitat richness (i.e., habitat richness corrected for actual cell area) ranged from 0 to 1.5 (Figure 1b).

Both maps show a heterogeneous distribution of habitat richness, peaking along the main mountain chains of Southern Europe and Central Europe as well as the Baltic area (Figure 1). Moreover, the habitat richness was positively correlated (*r* = .34, *p* < .001) with the richness of the species listed in the Annex species of the Birds and Habitats Directives as reported by every country (see Figure S2).

**FIGURE 2:**
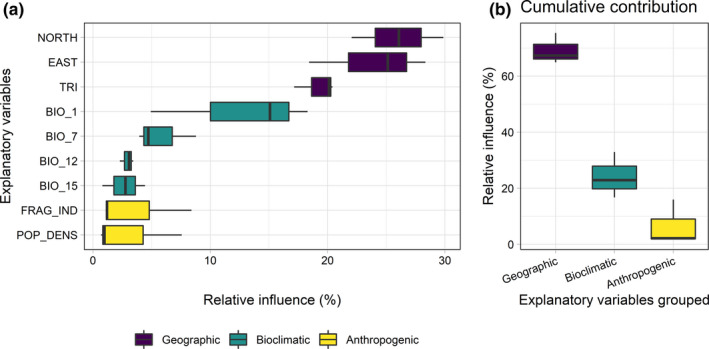
Boxplots showing percentages of relative influence and cumulative contribution of single and grouped explanatory variables in accounting for habitat richness. The contributions of variables were estimated across the GLM, GAM, and BRT models. Panel a: the relative influence of each explanatory variable. Panel b: the cumulative contribution for each group of the explanatory variables selected. Geographic variables: NORTH = northing, EAST = easting, TRI = terrain ruggedness index. Bioclimatic variables BIO_1 = mean annual temperature, BIO_7 = temperature annual range, BIO_12 = total annual precipitation, BIO_15 = precipitation seasonality. Anthropogenic variables: FRAG_IND = landscape fragmentation index, POP_DENS = human population density

Geographic variables showed similar effects across the different models, having the greatest cumulative contribution (Figure 2). Northing and Easting were the most important variables, with about 25% of relative influence each. Terrain ruggedness index (TRI), with about 20% of relative influence, was the third most important variable affecting habitat richness.

Climatic variables had widely different relative influence, with annual mean temperature having far more influence (ca. 15%) than the other three climatic variables taken together (annual temperature range, total annual precipitation, and precipitation seasonality; Figure 2). Finally, anthropogenic variables had only a minor contribution (ca. 5%) (Figure 2).

### 3.1 Effects of environmental variables on habitat richness

**FIGURE 3:**
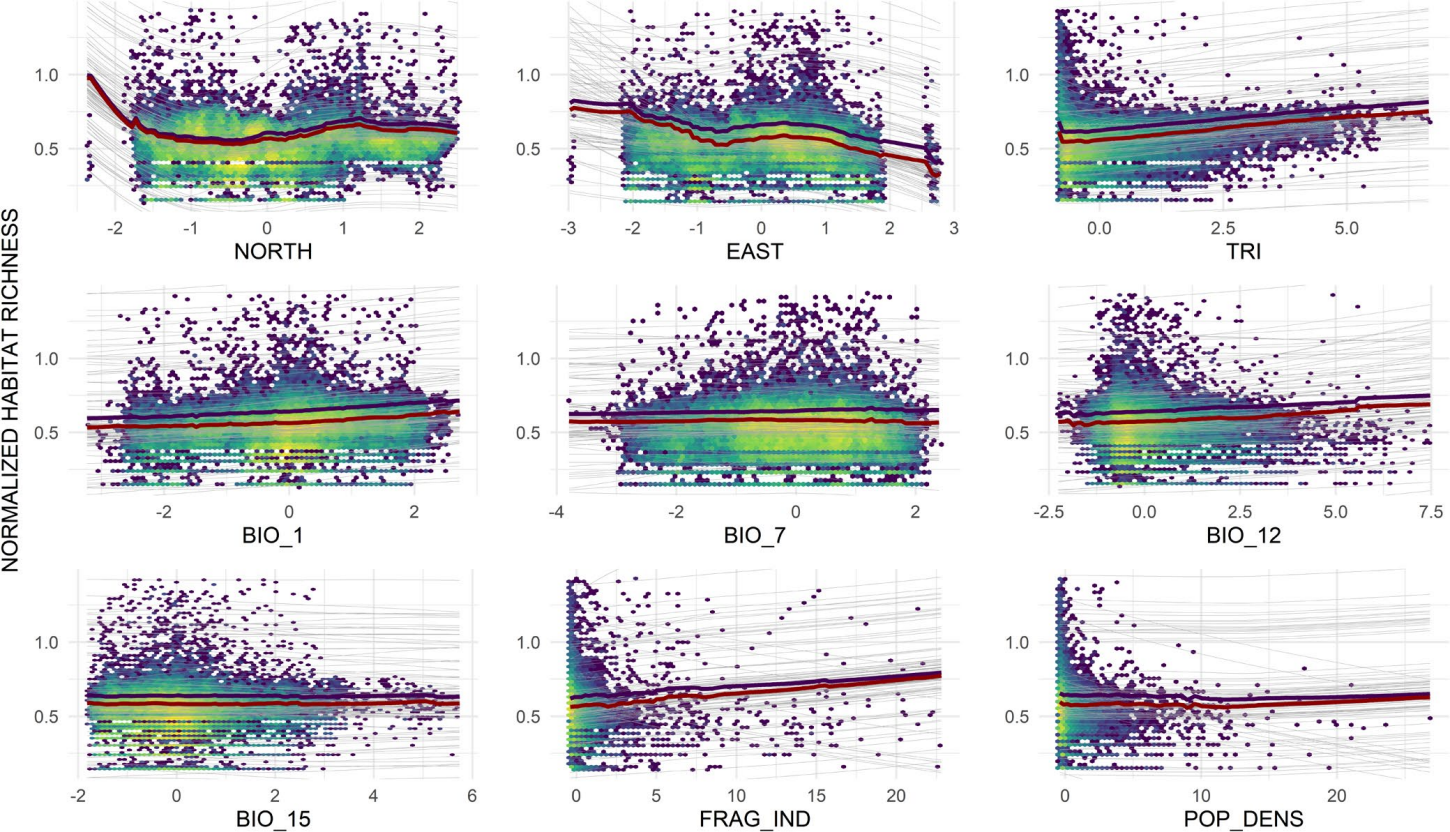
Relationships between explanatory variables and normalized habitat richness. The density of (10 km × 10 km) grid cells is indicated by hexagonal binning using the viridis color scale (varying from high density in yellow to low density in violet). Gray lines represent the 100 inflated response curves averaged across the three models used: generalized linear models (GLMs), generalized additive models (GAMs), and boosted regression trees (BRTs). Red lines are the median value, violet lines are the mean value of the inflated response curves. Geographic variables: NORTH = northing, EAST = easting, TRI = terrain ruggedness index. Bioclimatic variables: BIO_1 = mean annual temperature, BIO_7 = temperature annual range, BIO_12 = total annual precipitation, BIO_15 = precipitation seasonality. Anthropogenic variables: FRAG_IND = landscape fragmentation index, POP_DENS = human population density

**FIGURE 4:**
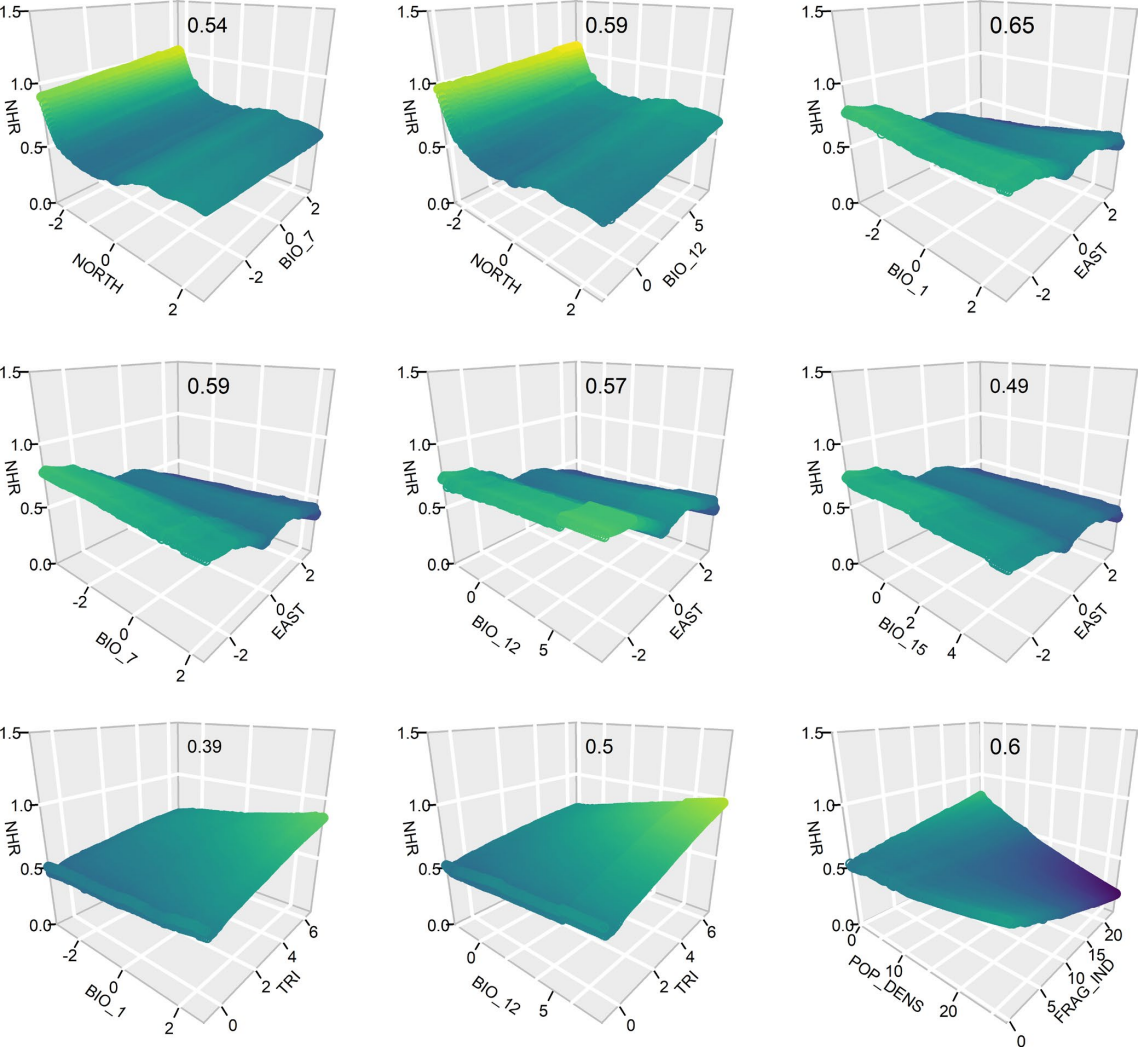
Surface plots show the interactions among the explanatory variables, x‐ and y‐axis represent pairs of explanatory variables and z‐axis is the magnitude of the interaction on the response variable. Only interactions above the given threshold (|*z*| = 0.3) are displayed. Geographic variables: TRI = terrain ruggedness index, NORTH = northing and EAST = easting. Bioclimatic variables: BIO_1 = mean annual temperature, BIO_7 = temperature annual range, BIO_12 = total annual precipitation, BIO_15= precipitation seasonality. Anthropogenic explanatory variables: FRAG_IND = landscape fragmentation index, POP_DENS = human population density

Both mean annual temperature and annual precipitation had a positive effect on normalized habitat richness, which also increased linearly with TRI (Figure 3). Habitat richness diminished toward eastern regions of the EU, while latitude showed an initial negative effect on habitat richness, it then slightly increased. The interactions between geographic and bioclimatic variables have shown a strong impact on habitat richness (Figure 4). The highest values of habitat richness were observed at low latitudes, where annual precipitation was moderately high. The positive trends observed for mean annual temperature and mean annual precipitation were considerably stronger in more rugged cells (higher TRI in Figure 4). Other interactions among bioclimatic variables did not show any remarkable trend. The landscape fragmentation index on its own showed a positive effect on habitat richness. In contrast, population density index did not reveal any clear pattern (Figure 3), though it has interesting interaction with landscape fragmentation values; in particular, high landscape fragmentation affects positively habitat richness at low population but negatively at high population density (Figure 4).

### 3.2 Model performance

The explained deviance of the GLMs without autocovariate was 0.22, whereas after accounting for spatial autocorrelation, the explained deviance reached 0.59 (Table 2). The root mean square error (RMSE) indicated large differences among the explained deviance by the GLM and GAM on one side and the BRT on the other side, with the former models showing 0.27 and 0.26, respectively, and the latter 0.85. By adding the autocovariates to the models, GLM and GAM showed substantial improvements (reducing the RMSE to 0.2 in each case), whereas the BRT did not.

**TABLE 2:** Explained deviance (*D* ^2^) and root mean square error (RMSE) of the models with and without autocovariate (“RAC”)

Model	*D* ^2^	RMSE
GLM	0.22	0.27
GLM with RAC	0.59	0.20
GAM	0.27	0.26
GAM with RAC	0.59	0.20
GBM	NA	0.85
GBM with RAC	NA	0.85

## 4 DISCUSSION

Our analysis shows that the relationship between habitat richness and the richness of species with conservation concern is positive and monotonic. Indeed, it is widely reported that a high number of habitats support a high number of species (Hortal et al., 2009) and vice versa (Jiménez‐Alfaro et al., 2014). This confirms that habitat diversity at continental scales can be used as a pragmatic proxy for species richness and is thus a useful tool to assess the status of biodiversity conservation (Kallimanis et al., 2008; Pyšek et al., 2002; Saura, 2021a). We also found that geographic variables are the most relevant variables to shape habitat richness, while the effects of bioclimatic and anthropogenic variables were less evident but still significant.

### 4.1 Geographic variables

Geographic variables showed the strongest association with habitat richness. We found that a major predictor of large‐scale habitat richness was latitude, which is considered a proxy for other environmental variables (e.g., solar radiation and productivity; Archibald et al., 2010, Qian & Ricklefs, 2011). This variable is widely used as a predictor of species richness and diversity (Gaston, 2007; Hillebrand, 2004). In particular, our findings support the general tendency of biodiversity to decrease from lower to higher latitudes (Fine, 2015; MacArthur, 1984; Stevens, 1989). At smaller scales (i.e., national), Drakou et al. (2011) report a positive relationship with habitat richness for both latitude and longitude. In our study, a weak decrease of habitat richness was observed for longitude, probably due to lower habitat richness of the eastern countries, due to continentality and likely as a result of varying completeness in reporting the full lists of habitat types for some SE countries.

Habitat richness was also positively correlated with topographic complexity. The importance of environmental heterogeneity in controlling biodiversity is widely recognized in ecological theory (Hjort et al., 2015; Huston, 1994; Marini et al., 2011; Stein et al., 2014). TRI can be considered one of the main factors contributing to explain habitat and species richness (López‐González et al., 2015; Stein et al., 2014; Tews et al., 2004), and its effect is expected to increase with spatial grain (Stein et al., 2014). Indeed, topographically complex areas offer a larger number and more different local conditions than topographically simple areas, leading to a higher number of habitat types packed into the same area (Stein et al., 2014). TRI contributes to species richness not only by providing an abundance of niches in space but also by offering relatively stable niches in time (Davies et al., 2007; Irl et al., 2015; Thuiller et al., 2006). Several studies showed an increase of species richness in relation to the increase of surface complexity (Cramer & Verboom, 2017; Farwell et al., 2021) and highly heterogeneous areas supporting more species than areas of lower heterogeneity (Hortal et al., 2009; Kallimanis et al., 2008; Rosenzweig, 1995).

### 4.2 Climate variable

Temperature and precipitation variables were the second most important group in explaining habitat richness. It is widely reported that climatic variables are considered as main drivers of broad‐scale patterns in species richness (Grytnes & McCain, 2007; Thuiller et al., 2004; Vetaas et al., 2019; Xu et al., 2014). We observed a positive association of habitat richness with annual precipitation as reported also for aquatic, coastal, and forest EU habitats (Drakou et al., 2011). Precipitation is very unevenly distributed across time (among and within years) and space in Europe (Rajah et al., 2014; Zolina, 2012). Due to climate changes, an increase in precipitation variability is expected in the near future, and this phenomenon could negatively affect both species and habitat diversity (Adler & Levine, 2007; Pearson & Carroll, 1998). Moreover, not only precipitation but other factors such as potential evapotranspiration (Adhikari et al., 2019) or soil water availability (Daws et al., 2002; Vetaas & Ferrer‐Castán, 2008) should be considered to better predict species and habitat distributions.

Among climate variables, mean annual temperature was the most strongly associated with habitat richness. Positive correlation among plant species richness and temperature is widely reported (Diogo et al., 2020; Gottfried et al., 2012), but this relationship may be reversed when water availability is limited (Pausas & Austin, 2001). Annual temperature range has a slight positive effect on habitat richness with a final slight decrease, highlighting the secondary role of temperature range in shaping species and habitat distribution (Austin & Niel, 2011). Interactions among climatic variables did not produce any strong effects as compared to the single bioclimatic variables. Instead, the interactions of mean annual temperature and mean annual precipitation with TRI suggest a positive effect on habitat richness of environmental heterogeneity with both the increase in temperature and precipitation.

### 4.3 Anthropogenic variables

Population density is considered one of the main proxies defining human pressure on nature (Sanderson et al., 2002; Venter et al., 2016). Among the anthropogenic variables considered, the LFI showed the strongest association with habitat richness, as already found by other authors (e.g., Jaeger, 2000). This anthropogenic factor breaks ecological interrelations between the habitat patches and decreases their ability to provide various ecosystem services (Jaeger, 2000).

The decrease of habitat richness along with increasing human pressure was not as strong as expected. Indeed, environmental factors are typically more important for explaining species richness than human impacts (Howard et al., 2020). Moreover, the weaker influence of anthropogenic variables was probably due to the coarse resolution used in this study (Curtis et al., 2018; Niemiec et al., 2018; Woodbridge et al., 2020).

On the other hand, the synergistic interaction between fragmentation and population density had a strongly negative influence on habitat richness, also affecting habitat conservation (Ewers & Didham, 2006). This finding suggests that the interaction of anthropogenic variables (Newbold et al., 2015) could be of greater importance than climate interactions (Holman et al., 2017; Lehsten et al., 2015). Thus, land‐use modification in the near future should be planned in order to decrease landscape fragmentation and increase habitat connectivity.

## 5 CONCLUSIONS AND IMPLICATIONS FOR CONSERVATION PLANNING

The evaluation of the variables associated with habitat richness here performed for the first time at the European continental scale revealed that geographic and climatic variables are more influential than anthropogenic variables for explaining habitat richness distribution. Despite not showing the strongest correlations, we found that human activities were indeed relevant in controlling the distribution of habitat richness, as the interaction among anthropogenic variables had a strong negative effect on habitat richness. Thus, for environmental management, it is important to consider the cumulative effect of interactions between natural and anthropogenic variables. Indeed, increasing human populations, long‐term land cover changes, and pressures from invasive alien species have all been linked to habitat transformation (Banks‐Leite et al., 2020) and biodiversity loss (Campagnaro et al., 2018; Cardillo et al., 2004; Chase et al., 2020; Davies et al., 2006; Leclère et al., 2020; Pacifici et al., 2017). Landscape transformation and habitat degradation outside protected areas may also contribute to landscape fragmentation leading to isolated “islands” with low connectivity (Chase et al., 2020; Keeley et al., 2018). The present study highlights how biodiversity policies of the EU, such as Habitats Directive, have a central role not only in biodiversity conservation (Gameiro et al., 2020) but also providing continental scale data which are fundamental to investigate biodiversity patterns, as already demonstrated by Hoffmann et al. (2018) in relation to the European protected area network. This finding has important implications for conservation planning through the use of European habitat inventories originally created for reporting regional status of the Natura 2000 network. Knowing the inherent vulnerability of some habitats could aid decisions regarding European conservation priorities and could form the basis for a biodiversity conservation roadmap (Arlidge et al., 2018).

## CONFLICT OF INTEREST

No conflict of interest.

## AUTHOR CONTRIBUTIONS

**Marco Cervellini:** Data curation (lead); Investigation (lead); Methodology (equal); Project administration (lead); Writing – original draft (lead); Writing – review & editing (equal). **Michele Di Musciano:** Formal analysis (lead); Investigation (lead); Methodology (equal); Writing – original draft (lead); Writing – review & editing (equal). **Piero Zannini:** Formal analysis (lead); Investigation (equal); Methodology (equal); Writing – original draft (equal); Writing – review & editing (equal). **Simone Fattorini:** Investigation (lead); Writing – review & editing (equal). **Borja Jiménez‐Alfaro:** Investigation (lead); Writing – review & editing (equal). **Emiliano Agrillo:** Investigation (equal); Writing – review & editing (equal). **Fabio Attorre:** Investigation (equal); Writing – review & editing (equal). **Pierangela Angelini:** Investigation (equal); Writing – review & editing (equal). **Carl Beierkuhnlein:** Investigation (equal); Writing – review & editing (equal). **Laura Casella:** Investigation (equal); Writing – review & editing (equal). **Richard Field:** Investigation (equal); Writing – review & editing (equal). **Jan‐Christopher Fischer:** Investigation (equal); Writing – review & editing (equal). **Piero Genovesi:** Investigation (equal); Writing – review & editing (equal). **Samuel Hoffmann:** Investigation (equal); Writing – review & editing (equal). **Severin D. H. Irl:** Investigation (equal); Writing – review & editing (equal). **Juri Nascimbene:** Investigation (equal); Writing – review & editing (equal). **Duccio Rocchini:** Investigation (equal); Writing – review & editing (equal). **Manuel Steinbauer:** Investigation (equal); Writing – review & editing (equal). **Ole R. Vetaas:** Investigation (equal); Writing – review & editing (equal). **Alessandro Chiarucci:** Conceptualization (lead); Investigation (equal); Writing – review & editing (equal).

## Supporting information

Figure S1

Figure S2

Figure S3

Figure S4

Supplementary figure captions

## Data Availability

The data that support the findings of this study are openly available in Dryad at https://doi.org/10.5061/dryad.m0cfxpp52

## References

[ece38409-bib-0001] Adhikari, A. , Mainali, K. P. , Rangwala, I. , & Hansen, A. J. (2019). Various measures of potential evapotranspiration have species‐specific impact on species distribution models. Ecological Modelling, 414, 108836. 10.1016/j.ecolmodel.2019.108836

[ece38409-bib-0002] Adler, P. B. , & Levine, J. M. (2007). Contrasting relationships between precipitation and species richness in space and time. Oikos, 116, 221–232. 10.1111/j.0030-1299.2007.15327.x

[ece38409-bib-0003] Alsterberg, C. , Roger, F. , Sundbäck, K. , Juhanson, J. , Hulth, S. , Hallin, S. , & Gamfeldt, L. (2017). Habitat diversity and ecosystem multifunctionality‐The importance of direct and indirect effects. Science Advances, 3, e1601475. 10.1126/sciadv.1601475 28246634 PMC5298852

[ece38409-bib-0004] Amatulli, G. , Domisch, S. , Tuanmu, M. N. , Parmentier, B. , Ranipeta, A. , Malczyk, J. , & Jetz, W. (2018). A suite of global, cross‐scale topographic variables for environmental and biodiversity modeling. Scientific Data, 5(1). 10.1038/sdata.2018.40 PMC5859920 29557978

[ece38409-bib-0005] Archibald, S. B. , Bossert, W. H. , Greenwood, D. R. , & Farrell, B. D. (2010). Seasonality, the latitudinal gradient of diversity, and Eocene insects. Paleobiology, 36(3), 374–398. 10.1666/09021.1

[ece38409-bib-0006] Arlidge, W. N. S. , Bull, J. W. , Addison, P. F. E. , Burgass, M. J. , Gianuca, D. , Gorham, T. M. , Jacob, C. , Shumway, N. , Sinclair, S. P. , Watson, J. E. M. , Wilcox, C. , & Milner‐Gulland, E. J. (2018). A global mitigation hierarchy for nature conservation. BioScience, 68, 336–347. 10.1093/biosci/biy029 29731513 PMC5925785

[ece38409-bib-0007] Arrhenius, O. (1921). Species and Area. The Journal of Ecology, 9(1), 95. 10.2307/2255763

[ece38409-bib-0008] Austin, M. P. , & Van Niel, K. P. (2011). Improving species distribution models for climate change studies: variable selection and scale. Journal of Biogeography, 38(1), 1–8. 10.1111/j.1365-2699.2010.02416.x

[ece38409-bib-0009] Banks‐Leite, C. , Ewers, R. M. , Folkard‐Tapp, H. , & Fraser, A. (2020). Countering the effects of habitat loss, fragmentation, and degradation through habitat restoration. One Earth, 3(6), 672–676. 10.1016/j.oneear.2020.11.016

[ece38409-bib-0010] Bardos, D. C. , Guillera‐Arroita, G. , & Wintle, B. A. (2015). Valid auto‐models for spatially autocorrelated occupancy and abundance data. Methods in Ecology and Evolution, 6, 1137–1149. 10.1111/2041-210X.12402

[ece38409-bib-0011] Bivand, R. S. , & Wong, D. W. (2018). Comparing implementations of global and local indicators of spatial association. Test, 27, 716–748. 10.1007/s11749-018-0599-x

[ece38409-bib-0012] Brandt, L. A. , Benscoter, A. M. , Harvey, R. , Speroterra, C. , Bucklin, D. , Romañach, S. S. , Watling, J. I. , & Mazzotti, F. J. (2017). Comparison of climate envelope models developed using expert‐selected variables versus statistical selection. Ecological Modelling, 345, 10–20. 10.1016/j.ecolmodel.2016.11.016

[ece38409-bib-0013] Brown, J. , & Lomolino, M. (2005). Biogeography. Sinauer.

[ece38409-bib-0014] Bunce, R. , Bogers, M. , Evans, D. , Halada, L. , Jongman, R. , Mucher, C. A. , Bauch, B. , de Blust, G. , Parr, T. W. , & Olsvig‐Whittaker, L. (2013). The significance of habitats as indicators of biodiversity and their links to species. Ecological Indicators, 33, 19–25. 10.1016/j.ecolind.2012.07.014

[ece38409-bib-0015] Calcagno, V. , & de Mazancourt, C. (2010). glmulti: An R package for easy automated model selection with (generalized) linear models. Journal of Statistical Software, 34(12). 10.18637/jss.v034.i12

[ece38409-bib-0016] Campagnaro, T. , Brundu, G. , & Sitzia, T. (2018). Five major invasive alien tree species in European Union forest habitat types of the Alpine and Continental biogeographical regions. Journal for Nature Conservation, 43, 227–238. 10.1016/j.jnc.2017.07.007

[ece38409-bib-0017] Cardillo, M. , Purvis, A. , Sechrest, W. , Gittleman, J. L. , Bielby, J. , & Mace, G. M. (2004). Human population density and extinction risk in the world’s carnivores. PLoS Biology, 2, e197. 10.1371/journal.pbio.0020197 15252445 PMC449851

[ece38409-bib-0018] Carnell, R. (2020). lhs: Latin hypercube samples. Retrieved from https://CRAN.R‐project.org/package=lhs

[ece38409-bib-0019] Chase, J. M. , Blowes, S. A. , Knight, T. M. , Gerstner, K. , & May, F. (2020). Ecosystem decay exacerbates biodiversity loss with habitat loss. Nature, 584, 238–243. 10.1038/s41586-020-2531-2 32728213

[ece38409-bib-0020] Cramer, M. D. , & Verboom, G. A. (2017). Measures of biologically relevant environmental heterogeneity improve prediction of regional plant species richness. Journal of Biogeography, 44, 579–591. 10.1111/jbi.12911

[ece38409-bib-0021] Crase, B. , Liedloff, A. C. , & Wintle, B. A. (2012). A new method for dealing with residual spatial autocorrelation in species distribution models. Ecography, 35, 879–888. 10.1111/j.1600-0587.2011.07138.x

[ece38409-bib-0022] Curtis, P. G. , Slay, C. M. , Harris, N. L. , Tyukavina, A. , & Hansen, M. C. (2018). Classifying drivers of global forest loss. Science, 361, 1108–1111. 10.1126/science.aau3445 30213911

[ece38409-bib-0023] Davies, C. E. , Dorian, M. , & Mark, O. H. (2004). EUNIS habitat classification revised 2004. Report to: European Environment Agency‐European Topic Centre on Nature Protection and Biodiversity (pp. 127–143).

[ece38409-bib-0024] Davies, R. G. , Orme, C. D. L. , Storch, D. , Olson, V. A. , Thomas, G. H. , Ross, S. G. , Ding, T. S. , Rasmussen, P. C. , Bennett, P. M. , Owens, I. P. F. , Blackburn, T. M. , & Gaston, K. J. (2007). Topography, energy and the global distribution of bird species richness. Proceedings of the Royal Society B: Biological Sciences, 274(1614), 1189–1197. 10.1098/rspb.2006.0061 PMC2189561 17311781

[ece38409-bib-0025] Davies, R. G. , Orme, C. D. L. , Olson, V. , Thomas, G. H. , Ross, S. G. , Ding, T.‐S. , Rasmussen, P. C. , Stattersfield, A. J. , Bennett, P. M. , Blackburn, T. M. , Owens, I. P. F. , & Gaston, K. J. (2006). Human impacts and the global distribution of extinction risk. Proceedings of the Royal Society B: Biological Sciences, 273, 2127–2133. 10.1098/rspb.2006.3551 PMC1635517 16901831

[ece38409-bib-0026] Daws, M. I. , Mullins, C. E. , Burslem, D. F. R. P. , Paton, S. R. , & Dalling, J. W. (2002). Plant and Soil, 238(1), 79–89. 10.1023/a:1014289930621

[ece38409-bib-0027] Dianzinga, N. T. , Moutoussamy, M. L. , Sadeyen, J. , Ravaomanarivo, L. H. R. , & Frago, E. (2020). The interacting effect of habitat amount, habitat diversity and fragmentation on insect diversity along elevational gradients. Journal of Biogeography, 47(11), 2377–2391. 10.1111/jbi.13959

[ece38409-bib-0028] Diogo, I. J. S. , Santos, K. , Costa, I. R. , & Santos, F. A. M. (2020). Effects of topography and climate on Neotropical mountain forests structure in the semiarid region. Applied Vegetation Science, 24, 1–12. 10.1111/avsc.12527

[ece38409-bib-0029] Dormann, C. F. , Elith, J. , Bacher, S. , Buchmann, C. , Carl, G. , Carré, G. , Marquéz, J. R. G. , Gruber, B. , Lafourcade, B. , Leitão, P. J. , Münkemüller, T. , McClean, C. , Osborne, P. E. , Reineking, B. , Schröder, B. , Skidmore, A. K. , Zurell, D. , & Lautenbach, S. (2013). Collinearity: A review of methods to deal with it and a simulation study evaluating their performance. Ecography, 36, 27–46. 10.1111/j.1600-0587.2012.07348.x

[ece38409-bib-0030] Drakou, E. G. , Kallimanis, A. S. , Mazaris, A. D. , Apostolopoulou, E. , & Pantis, J. D. (2011). Habitat type richness associations with environmental variables: A case study in the Greek Natura 2000 aquatic ecosystems. Biodiversity and Conservation, 20, 929–943. 10.1007/s10531-011-0005-4

[ece38409-bib-0031] Dufour, A. , Gadallah, F. , Wagner, H. H. , Guisan, A. , & Buttler, A. (2006). Plant species richness and environmental heterogeneity in a mountain landscape: Effects of variability and spatial configuration. Ecography, 29, 573–584. 10.1111/j.0906-7590.2006.04605.x

[ece38409-bib-0032] Elith, J. , H. Graham, C. , P. Anderson, R. , Dudík, M. , Ferrier, S. , Guisan, A. , J. Hijmans, R. , Huettmann, F. , R. Leathwick, J. , Lehmann, A. , Li, J. , G. Lohmann, L. , A. Loiselle, B. , Manion, G. , Moritz, C. , Nakamura, M. , Nakazawa, Y. , McC. M. Overton, J. , Townsend Peterson, A. , … E. Zimmermann, N. (2006). Novel methods improve prediction of species’ distributions from occurrence data. Ecography, 29, 129–151. 10.1111/j.2006.0906-7590.04596.x

[ece38409-bib-0033] EU‐DEM . (2021). Copernicus land monitoring service. Retrieved from https://land.copernicus.eu/imagery‐in‐situ/eu‐dem

[ece38409-bib-0034] European Commission . (2021). Natura 2000 in the Marine Environment – Appendix 2 of the “Lists of existing marine Habitat types and Species for different Member States”. Retrieved from https://ec.europa.eu/environment/nature/natura2000/marine/index_en.htm

[ece38409-bib-0035] European Environment Agency (EEA) . (2009). Raster data on population density using Corine Land Cover 2000 inventory. Retrieved from https://www.eea.europa.eu/data‐and‐maps/data/population‐density‐disaggregated‐with‐corine‐land‐cover‐2000‐2

[ece38409-bib-0036] European Environment Agency (EEA) . (2011). Landscape fragmentation in Europe. Retrieved from https://www.eea.europa.eu/data‐and‐maps/data/landscape‐fragmentation‐indicator‐effective‐mesh

[ece38409-bib-0037] European Environment Agency (EEA) . (2013). EEA reference grid. Retrieved from https://www.eea.europa.eu/data‐and‐maps/data/eea‐reference‐grids‐2

[ece38409-bib-0038] European Environment Agency (EEA) . (2020). Article 17–2015 dataset – III report of Habitat distribution and of species occurrence data. Retrieved from https://www.eea.europa.eu/data‐and‐maps/data/article‐17‐database‐habitats‐directive‐92‐43‐eec‐2

[ece38409-bib-0039] European Environment Agency (EEA) . (2020). Article 12–2015 spatial data – III report of Bird Directive species occurrence data. Retrieved from https://www.eea.europa.eu/data‐and‐maps/data/article‐12‐database‐birds‐directive‐2009‐147‐ec‐1

[ece38409-bib-0040] Ewers, R. M. , & Didham, R. K. (2006). Confounding factors in the detection of species responses to habitat fragmentation. Biological Reviews, 81, 117–142. 10.1017/S1464793105006949 16318651

[ece38409-bib-0041] Fahrig, L. (2013). Rethinking patch size and isolation effects: The habitat amount hypothesis. Journal of Biogeography, 40, 1649–1663. 10.1111/jbi.12130

[ece38409-bib-0042] Fahrig, L. (2021). What the habitat amount hypothesis does and does not predict: A reply to Saura. Journal of Biogeography, 48, 1530–1535. 10.1111/jbi.14061

[ece38409-bib-0043] Farwell, L. S. , Gudex‐Cross, D. , Anise, I. E. , Bosch, M. J. , Olah, A. M. , Radeloff, V. C. , Razenkova, E. , Rogova, N. , Silveira, E. M. O. , Smith, M. M. , & Pidgeon, A. M. (2021). Satellite image texture captures vegetation heterogeneity and explains patterns of bird richness. Remote Sensing of Environment, 253, 112175. 10.1016/j.rse.2020.112175

[ece38409-bib-0044] Fick, S. E. , & Hijmans, R. J. (2017). WorldClim 2: New 1‐km spatial resolution climate surfaces for global land areas. International Journal of Climatology, 37, 4302–4315. 10.1002/joc.5086

[ece38409-bib-0045] Field, R. , Hawkins, B. A. , Cornell, H. V. , Currie, D. J. , Diniz‐Filho, J. A. F. , Guégan, J.‐F. , Kaufman, D. M. , Kerr, J. T. , Mittelbach, G. G. , Oberdorff, T. , O’Brien, E. M. , & Turner, J. R. G. (2009). Spatial species‐richness gradients across scales: A meta‐analysis. Journal of Biogeography, 36, 132–147. 10.1111/j.1365-2699.2008.01963.x

[ece38409-bib-0046] Fine, P. V. A. (2015). Ecological and evolutionary drivers of geographic variation in species diversity. Annual Review of Ecology, Evolution, and Systematics, 46, 369–392.

[ece38409-bib-0047] Fletcher, R. , & Fortin, M. J. (2018). Accounting for spatial dependence in ecological data. Spatial ecology and conservation modeling (1st ed., pp. 169–210). Springer, Cham.

[ece38409-bib-0136] Gallego, F. J. (2010). A population density grid of the European Union. Population and Environment, 31(6), 460–473.

[ece38409-bib-0048] Gameiro, J. , Silva, J. P. , Franco, A. M. A. , & Palmeirim, J. M. (2020). Effectiveness of the European Natura 2000 network at protecting Western Europe’s agro‐steppes. Biological Conservation, 248, 108681. 10.1016/j.biocon.2020.108681

[ece38409-bib-0049] Gao, J. , & Liu, Y. (2018). Climate stability is more important than water‐energy variables in shaping the elevational variation in species richness. Ecology and Evolution, 8, 6849–7245. 10.1002/ece3.4202 PMC6065338 30073051

[ece38409-bib-0050] Gaston, K. J. (2000). Global patterns in biodiversity. Nature, 405, 220–227. 10.1038/35012228 10821282

[ece38409-bib-0051] Gaston, K. J. (2007). Latitudinal gradient in species richness. Current Biology, 17, R574. 10.1016/j.cub.2007.05.013 17686422

[ece38409-bib-0052] Gibb, R. , Redding, D. W. , Chin, K. Q. , Donnelly, C. A. , Blackburn, T. M. , Newbold, T. , & Jones, K. E. (2020). Zoonotic host diversity increases in human‐dominated ecosystems. Nature, 584, 398–402. 10.1038/s41586-020-2562-8 32759999

[ece38409-bib-0053] Gottfried, M. , Pauli, H. , Futschik, A. , Akhalkatsi, M. , Barančok, P. , Benito Alonso, J. L. , Coldea, G. , Dick, J. , Erschbamer, B. , Fernández Calzado, M. R. , Kazakis, G. , Krajči, J. , Larsson, P. , Mallaun, M. , Michelsen, O. , Moiseev, D. , Moiseev, P. , Molau, U. , Merzouki, A. , … Grabherr, G. (2012). Continent‐wide response of mountain vegetation to climate change. Nature Climate Change, 2, 111–115. 10.1038/nclimate1329

[ece38409-bib-0054] Grytnes, J. A. , & McCain, C. M. (2007). Elevational trends in biodiversity. Encyclopedia of Biodiversity, 2, 1–8.

[ece38409-bib-0055] Hall, L. S. , Krausman, P. R. , & Morrison, M. L. (1997). The habitat concept and a plea for standard terminology. Wildlife Society Bulletin, 25, 173–182.

[ece38409-bib-0056] Harisena, N. V. , Groen, T. A. , Toxopeus, A. G. , & Naimi, B. (2021). When is variable importance estimation in species distribution modelling affected by spatial correlation? Ecography, 44(5), 778–788. 10.1111/ecog.05534

[ece38409-bib-0057] Heidrich, L. , Bae, S. , Levick, S. , Seibold, S. , Weisser, W. , Krzystek, P. , Magdon, P. , Nauss, T. , Schall, P. , Serebryanyk, A. , Wöllauer, S. , Ammer, C. , Bässler, C. , Doerfler, I. , Fischer, M. , Gossner, M. M. , Heurich, M. , Hothorn, T. , Jung, K. , … Müller, J. (2020). Heterogeneity–diversity relationships differ between and within trophic levels in temperate forests. Nature Ecology & Evolution, 4, 1204–1212. 10.1038/s41559-020-1245-z 32661404

[ece38409-bib-0058] Hickey, J. (2016). gbm3: Generalized boosted regression models. Retrieved from https://github.com/gbm‐developers/gbm3

[ece38409-bib-0059] Hillebrand, H. (2004). On the generality of the latitudinal diversity gradient. The American Naturalist, 163, 192–211. 10.1086/381004 14970922

[ece38409-bib-0060] Hjort, J. , Gordon, J. E. , Gray, M. , & Hunter, M. L. (2015). Why geodiversity matters in valuing nature’s stage: Why Geodiversity Matters. Conservation Biology, 29, 630–639. 10.1111/cobi.12510 25923307

[ece38409-bib-0061] Hoffmann, S. , Beierkuhnlein, C. , Field, R. , Provenzale, A. , & Chiarucci, A. (2018). Uniqueness of protected areas for conservation strategies in the European Union. Scientific Reports, 8, 6445. 10.1038/s41598-018-24390-3 29691423 PMC5915414

[ece38409-bib-0062] Holman, I. P. , Brown, C. , Janes, V. , & Sandars, D. (2017). Can we be certain about future land use change in Europe? A multi‐scenario, integrated‐assessment analysis. Agricultural Systems, 151, 126–135. 10.1016/j.agsy.2016.12.001 28163353 PMC5268336

[ece38409-bib-0063] Hortal, J. , Triantis, K. A. , Meiri, S. , Thébault, E. , & Sfenthourakis, S. (2009). Island species richness increases with habitat diversity. The American Naturalist, 174, E205–E217. 10.1086/645085 19857159

[ece38409-bib-0064] Howard, C. , Flather, C. H. , & Stephens, P. A. (2020). A global assessment of the drivers of threatened terrestrial species richness. Nature Communications, 11, 993. 10.1038/s41467-020-14771-6 PMC7033199 32080191

[ece38409-bib-0065] Huston, M. A. (1994). Biological diversity: The coexistence of species. Cambridge University Press.

[ece38409-bib-0066] Irl, S. D. H. , Harter, D. E. V. , Steinbauer, M. J. , Gallego Puyol, D. , Fernández‐Palacios, J. M. , Jentsch, A. , & Beierkuhnlein, C. (2015). Climate vs. topography–spatial patterns of plant species diversity and endemism on a high‐elevation island. Journal of Ecology, 103, 1621–1633. 10.1111/1365-2745.12463

[ece38409-bib-0067] Jaeger, J. A. G. (2000). Landscape division, splitting index, and effective mesh size: New measures of landscape fragmentation. Landscape Ecology, 15, 115–130.

[ece38409-bib-0068] Jiménez‐Alfaro, B. , Chytrý, M. , Mucina, L. , Grace, J. B. , & Rejmánek, M. (2016). Disentangling vegetation diversity from climate‐energy and habitat heterogeneity for explaining animal geographic patterns. Ecology and Evolution, 6, 1515–1526. 10.1002/ece3.1972 26900451 PMC4747316

[ece38409-bib-0069] Jiménez‐Alfaro, B. , Chytrý, M. , Rejmánek, M. , & Mucina, L. (2014). The number of vegetation types in European countries: Major determinants and extrapolation to other regions. Journal of Vegetation Science, 25, 863–872. 10.1111/jvs.12145

[ece38409-bib-0070] Jung, M. , Dahal, P. R. , Butchart, S. H. M. , Donald, P. F. , De Lamo, X. , Lesiv, M. , Kapos, V. , Rondinini, C. , & Visconti, P. (2020). A global map of terrestrial habitat types. Scientific Data, 7, 256. 10.1038/s41597-020-00599-8 32759943 PMC7406504

[ece38409-bib-0071] Kallimanis, A. S. , Mazaris, A. D. , Tzanopoulos, J. , Halley, J. M. , Pantis, J. D. , & Sgardelis, S. P. (2008). How does habitat diversity affect the species–area relationship? Global Ecology and Biogeography, 17, 532–538. 10.1111/j.1466-8238.2008.00393.x

[ece38409-bib-0072] Kearney, M. (2006). Habitat, environment and niche: What are we modelling? Oikos, 115, 186–191.

[ece38409-bib-0073] Keeley, A. T. H. , Ackerly, D. D. , Cameron, D. R. , Heller, N. E. , Huber, P. R. , Schloss, C. A. , Thorne, J. H. , & Merenlender, A. M. (2018). New concepts, models, and assessments of climate‐wise connectivity. Environmental Research Letters, 13(7), 073002. 10.1088/1748-9326/aacb85

[ece38409-bib-0074] Keith, D. A. , Rodríguez, J. P. , Rodríguez‐Clark, K. M. , Nicholson, E. , Aapala, K. , Alonso, A. , Asmussen, M. , Bachman, S. , Basset, A. , Barrow, E. G. , Benson, J. S. , Bishop, M. J. , Bonifacio, R. , Brooks, T. M. , Burgman, M. A. , Comer, P. , Comín, F. A. , Essl, F. , Faber‐Langendoen, D. , … Zambrano‐Martínez, S. (2013). Scientific foundations for an IUCN red list of ecosystems. PLoS One, 8, e62111. 10.1371/journal.pone.0062111 23667454 PMC3648534

[ece38409-bib-0075] Keppel, G. , Gillespie, T. W. , Ormerod, P. , & Fricker, G. A. (2016). Habitat diversity predicts orchid diversity in the tropical south‐west Pacific. Journal of Biogeography, 43, 2332–2342. 10.1111/jbi.12805

[ece38409-bib-0076] Kerr, J. T. , & Packer, L. (1997). Habitat heterogeneity as a determinant of mammal species richness in high‐energy regions. Nature, 385, 252–254. 10.1038/385252a0

[ece38409-bib-0077] Leclère, D. , Obersteiner, M. , Barrett, M. , Butchart, S. H. M. , Chaudhary, A. , De Palma, A. , DeClerck, F. A. J. , Di Marco, M. , Doelman, J. C. , Dürauer, M. , Freeman, R. , Harfoot, M. , Hasegawa, T. , Hellweg, S. , Hilbers, J. P. , Hill, S. L. L. , Humpenöder, F. , Jennings, N. , Krisztin, T. , Mace, G. M. , Ohashi, H. , Popp, A. , Purvis, A. , Schipper, A. M. , Tabeau, A. , Valin, H. , van Meijl, H. , van Zeist, W. J. , Visconti, P. , Alkemade, R. , Almond, R. , Bunting, G. , Burgess, N. D. , Cornell, S. E. , Di Fulvio, F. , Ferrier, S. , Fritz, S. , Fujimori, S. , Grooten, M. , Harwood, T. , Havlík, P. , Herrero, M. , Hoskins, A. J. , Jung, M. , Kram, T. , Lotze‐Campen, H. , Matsui, T. , Meyer, C. , Nel, D. , Newbold, T. , Schmidt‐Traub, G. , Stehfest, E. , Strassburg, B. B. N. , van Vuuren, D. P. , Ware, C. , Watson, J. E. M. , Wu, W. , & Young, L. (2020). Bending the curve of terrestrial biodiversity needs an integrated strategy. Nature, 585(7826), 551–556. 10.1038/s41586-020-2705-y 32908312

[ece38409-bib-0078] Lehsten, V. , Sykes, M. T. , Scott, A. V. , Tzanopoulos, J. , Kallimanis, A. , Mazaris, A. , Verburg, P. H. , Schulp, C. J. E. , Potts, S. G. , & Vogiatzakis, I. (2015). Disentangling the effects of land‐use change, climate and CO_2_ on projected future European habitat types. Global Ecology and Biogeography, 24(6), 653–663. 10.1111/geb.12291

[ece38409-bib-0079] Liu, Q. , Buyantuev, A. , Wu, J. , Niu, J. , Yu, D. , & Zhang, Q. (2018). Intensive land‐use drives regional‐scale homogenization of plant communities. Science of the Total Environment, 644, 806–814. 10.1016/j.scitotenv.2018.07.019 29990929

[ece38409-bib-0080] Lomolino, M. V. (2000). Ecology’s most general, yet protean 1 pattern: The species‐area relationship. Journal of Biogeography, 27, 17–26.

[ece38409-bib-0081] López‐González, C. , Presley, S. J. , Lozano, A. , Stevens, R. D. , & Higgins, C. L. (2015). Ecological biogeography of Mexican bats: The relative contributions of habitat heterogeneity, beta diversity, and environmental gradients to species richness and composition patterns. Ecography, 38(3), 261–272. 10.1111/ecog.00813

[ece38409-bib-0082] Lüdecke, D. (2018). ggeffects: Tidy data frames of marginal effects from regression models. Journal of Open Source Software, 3(26), 772. 10.21105/joss.00772

[ece38409-bib-0083] MacArthur, R. H. (1984). Geographical ecology: Patterns in the distribution of species. Princeton University Press.

[ece38409-bib-0084] Malavasi, M. , Santoro, R. , Cutini, M. , Acosta, A. , & Carranza, M. L. (2016). The impact of human pressure on landscape patterns and plant species richness in Mediterranean coastal dunes. Plant Biosystems, 150, 73–82. 10.1080/11263504.2014.913730

[ece38409-bib-0085] Mantyka‐pringle, C. S. , Martin, T. G. , & Rhodes, J. R. (2012). Interactions between climate and habitat loss effects on biodiversity: A systematic review and meta‐analysis. Global Change Biology, 18, 1239–1252. 10.1111/j.1365-2486.2011.02593.x

[ece38409-bib-0086] Mantyka‐Pringle, C. S. , Martin, T. G. , & Rhodes, J. R. (2013). Interactions between climate and habitat loss effects on biodiversity: A systematic review and meta‐analysis. Global Change Biology, 19, 1642–1644. 10.1111/gcb.12148

[ece38409-bib-0087] Márcia Barbosa, A. , Real, R. , Muñoz, A.‐R. , & Brown, J. A. (2013). New measures for assessing model equilibrium and prediction mismatch in species distribution models. Diversity and Distributions, 19, 1333–1338. 10.1111/ddi.12100

[ece38409-bib-0088] Marini, L. , Bona, E. , Kunin, W. E. , & Gaston, K. J. (2011). Exploring anthropogenic and natural processes shaping fern species richness along elevational gradients. Journal of Biogeography, 38, 78–88. 10.1111/j.1365-2699.2010.02376.x

[ece38409-bib-0089] Meijer, J. R. , Huijbregts, M. A. J. , Schotten, K. C. G. J. , & Schipper, A. M. (2018). Global patterns of current and future road infrastructure. Environmental Research Letters, 13, 064006. 10.1088/1748-9326/aabd42

[ece38409-bib-0090] Mitchell, S. C. (2005). How useful is the concept of habitat? A critique. Oikos, 110, 634–638. 10.1111/j.0030-1299.2005.13810.x

[ece38409-bib-0091] Moser, D. , Dullinger, S. , Englisch, T. , Niklfeld, H. , Plutzar, C. , Sauberer, N. , Zechmeister, H. G. , & Grabherr, G. (2005). Environmental determinants of vascular plant species richness in the Austrian Alps: Plant species richness in the Alps. Journal of Biogeography, 32, 1117–1127. 10.1111/j.1365-2699.2005.01265.x

[ece38409-bib-0092] Mücher, C. A. , Hennekens, S. M. , Bunce, R. G. H. , Schaminée, J. H. J. , & Schaepman, M. E. (2009). Modelling the spatial distribution of Natura 2000 habitats across Europe. Landscape and Urban Planning, 92, 148–159. 10.1016/j.landurbplan.2009.04.003

[ece38409-bib-0093] Newbold, T. , Hudson, L. N. , Hill, S. L. L. , Contu, S. , Lysenko, I. , Senior, R. A. , Börger, L. , Bennett, D. J. , Choimes, A. , Collen, B. , Day, J. , De Palma, A. , Díaz, S. , Echeverria‐Londoño, S. , Edgar, M. J. , Feldman, A. , Garon, M. , Harrison, M. L. K. , Alhusseini, T. , Ingram, D. J. , Itescu, Y. , Kattge, J. , Kemp, V. , Kirkpatrick, L. , Kleyer, M. , Correia, D. L. P. , Martin, C. D. , Meiri, S. , Novosolov, M. , Pan, Y. , Phillips, H. R. P. , Purves, D. W. , Robinson, A. , Simpson, J. , Tuck, S. L. , Weiher, E. , White, H. J. , Ewers, R. M. , Mace, G. M. , Scharlemann, J. P. W. , & Purvis, A. (2015). Global effects of land use on local terrestrial biodiversity. Nature, 520(7545), 45–50. 10.1038/nature14324 25832402

[ece38409-bib-0094] Niemiec, R. M. , Asner, G. P. , Brodrick, P. G. , Gaertner, J. A. , & Ardoin, N. M. (2018). Scale‐dependence of environmental and socioeconomic drivers of albizia invasion in Hawaii. Landscape and Urban Planning, 169, 70–80. 10.1016/j.landurbplan.2017.08.008

[ece38409-bib-0095] Pacifici, M. , Visconti, P. , Butchart, S. H. M. , Watson, J. E. M. , Cassola, F. M. , & Rondinini, C. (2017). Species’ traits influenced their response to recent climate change. Nature Climate Change, 7, 205–208. 10.1038/nclimate3223

[ece38409-bib-0096] Pausas, J. G. , & Austin, M. P. (2001). Patterns of plant species richness in relation to different environments: An appraisal. Journal of Vegetation Science, 12, 153–166. 10.2307/3236601

[ece38409-bib-0097] Pearson, D. L. , & Carroll, S. S. (1998). Global patterns of species richness: Spatial models for conservation planning using bioindicator and precipitation data. Conservation Biology, 12, 809–821. 10.1046/j.1523-1739.1998.96460.x

[ece38409-bib-0098] Pearson, E. S. (1931). The test of significance for the correlation coefficient. Journal of the American Statistical Association, 26, 128–134. 10.1080/01621459.1931.10503208

[ece38409-bib-0099] Pedersen, T. L. (2020). patchwork: The composer of plots. Retrieved from https://CRAN.R‐project.org/package=patchwork

[ece38409-bib-0100] Pyšek, P. , Kučera, T. , & Jarošík, V. (2002). Plant species richness of nature reserves: the interplay of area, climate and habitat in a central European landscape. Global Ecology and Biogeography, 11(4), 279–289. 10.1046/j.1466-822x.2002.00288.x 32336944 PMC7165707

[ece38409-bib-0101] Qian, H. , & Ricklefs, R. E. (2011). Latitude, tree species diversity and the metabolic theory of ecology. Global Ecology and Biogeography, 20, 362–365.

[ece38409-bib-0102] Quintero, I. , & Jetz, W. (2018). Global elevational diversity and diversification of birds. Nature, 555, 246–250. 10.1038/nature25794 29466335

[ece38409-bib-0103] R Core Team . (2020). R: A language and environment for statistical computing. R Foundation for Statistical Computing. Retrieved from https://www.R‐project.org/

[ece38409-bib-0104] Radeloff, V. C. , Dubinin, M. , Coops, N. C. , Allen, A. M. , Brooks, T. M. , Clayton, M. K. , Costa, G. C. , Graham, C. H. , Helmers, D. P. , Ives, A. R. , Kolesov, D. , Pidgeon, A. M. , Rapacciuolo, G. , Razenkova, E. , Suttidate, N. , Young, B. E. , Zhu, L. , & Hobi, M. L. (2019). The dynamic habitat indices (dhis) from modis and global biodiversity. Remote Sensing of Environment, 222, 204–214. 10.1016/j.rse.2018.12.009

[ece38409-bib-0105] Rajah, K. , O'Leary, T. , Turner, A. , Petrakis, G. , Leonard, M. , & Westra, S. (2014). Changes to the temporal distribution of daily precipitation: Changing precipitation temporal patterns. Geophysical Research Letters, 41, 8887–8894. 10.1002/2014GL062156

[ece38409-bib-0106] Riley, S. J. , DeGloria, S. D. , & Elliot, R. (1999). A terrain ruggedness index that quantifies topographic heterogeneity. Intermountain Journal of Sciences, 5(1–4), 23–27.

[ece38409-bib-0107] Rosenzweig, M. L. (1995). Species diversity in space and time. Cambridge University Press.

[ece38409-bib-0108] Sanderson, E. W. , Jaiteh, M. , Levy, M. A. , Redford, K. H. , Wannebo, A. V. , & Woolmer, G. (2002). The human footprint and the last of the wild. BioScience, 52, 891.

[ece38409-bib-0109] Saura, S. (2021a). The Habitat Amount Hypothesis implies negative effects of habitat fragmentation on species richness. Journal of Biogeography, 48, 11–22. 10.1111/jbi.13958

[ece38409-bib-0110] Saura, S. (2021b). The habitat amount hypothesis predicts that fragmentation poses a threat to biodiversity: A reply to Fahrig. Journal of Biogeography, 48, 1536–1540. 10.1111/jbi.14122

[ece38409-bib-0111] Schloerke, B. , Cook, D. , Larmarange, J. , Briatte, F. , Marbach, M. , Thoen, E. , Elberg, A. , Toomet, O. , Crowley, J. , Hofmann, H. , & Wickham, H. (2021). GGally: Extension to “ggplot2”. Retrieved from https://CRAN.R‐project.org/package=GGally

[ece38409-bib-0112] Schmiedel, I. , & Culmsee, H. (2016). The influence of landscape fragmentation, expressed by the ‘Effective Mesh Size Index’, on regional patterns of vascular plant species richness in Lower Saxony, Germany. Landscape and Urban Planning, 153, 209–220. 10.1016/j.landurbplan.2016.01.012

[ece38409-bib-0113] Soetaert, K. (2019). Plot3D: Plotting multi‐dimensional data. Retrieved from https://CRAN.R‐project.org/package=plot3D

[ece38409-bib-0114] Stein, A. , Gerstner, K. , & Kreft, H. (2014). Environmental heterogeneity as a universal driver of species richness across taxa, biomes and spatial scales. Ecology Letters, 17(7), 866–880. 10.1111/ele.12277 24751205

[ece38409-bib-0115] Stevens, G. C. (1989). The latitudinal gradient in geographical range: How so many species coexist in the tropics. The American Naturalist, 133, 240–256. 10.1086/284913

[ece38409-bib-0116] Tews, J. , Brose, U. , Grimm, V. , Tielbörger, K. , Wichmann, M. C. , Schwager, M. , & Jeltsch, F. (2004). Animal species diversity driven by habitat heterogeneity/diversity: The importance of keystone structures. Journal of Biogeography, 31, 79–92. 10.1046/j.0305-0270.2003.00994.x

[ece38409-bib-0117] Thuiller, W. , Araújo, M. B. , & Lavorel, S. (2004). Do we need land‐cover data to model species distributions in Europe?: Do land‐cover data improve bioclimatic models? Journal of Biogeography, 31, 353–361. 10.1046/j.0305-0270.2003.00991.x

[ece38409-bib-0118] Thuiller, W. , F. Midgley, G. , Rougeti, M. , & M. Cowling, R. (2006). Predicting patterns of plant species richness in megadiverse South Africa. Ecography, 29, 733–744. 10.1111/j.0906-7590.2006.04674.x

[ece38409-bib-0119] Thuiller, W. , Lavorel, S. , Araujo, M. B. , Sykes, M. T. , & Prentice, I. C. (2005). Climate change threats to plant diversity in Europe. Proceedings of the National Academy of Sciences of the United States of America, 102, 8245–8250. 10.1073/pnas.0409902102 15919825 PMC1140480

[ece38409-bib-0120] Triantis, K. A. , Guilhaumon, F. , & Whittaker, R. J. (2012). The island species–area relationship: Biology and statistics. Journal of Biogeography, 39, 215–231. 10.1111/j.1365-2699.2011.02652.x

[ece38409-bib-0121] Triantis, K. A. , Vardinoyannis, K. , Tsolaki, E. P. , Botsaris, I. , Lika, K. , & Mylonas, M. (2006). Re‐approaching the small island effect. Journal of Biogeography, 33, 914–923. 10.1111/j.1365-2699.2006.01464.x

[ece38409-bib-0122] Tuanmu, M. , & Jetz, W. (2015). A global, remote sensing‐based characterization of terrestrial habitat heterogeneity for biodiversity and ecosystem modelling. Global Ecology and Biogeography, 24, 1329–1339.

[ece38409-bib-0123] Venter, O. , Sanderson, E. W. , Magrach, A. , Allan, J. R. , Beher, J. , Jones, K. R. , Possingham, H. P. , Laurance, W. F. , Wood, P. , Fekete, B. M. , Levy, M. A. , & Watson, J. E. M. (2016). Global terrestrial Human Footprint maps for 1993 and 2009. Scientific Data, 3, 160067. 10.1038/sdata.2016.67 27552448 PMC5127486

[ece38409-bib-0124] Vetaas, O. R. , Paudel, K. P. , & Christensen, M. (2019). Principal factors controlling biodiversity along an elevation gradient: Water, energy and their interaction. Journal of Biogeography, 46(8), 1652–1663. 10.1111/jbi.13564

[ece38409-bib-0125] Vetaas, O. R. , & Ferrer‐Castán, D. (2008). Patterns of woody plant species richness in the Iberian Peninsula: Environmental range and spatial scale. Journal of Biogeography, 35, 1863–1878. 10.1111/j.1365-2699.2008.01931.x

[ece38409-bib-0126] Wickham, H. (2016). ggplot2: Elegant graphics for data analysis. Springer.

[ece38409-bib-0127] Wickham, H. , François, R. , Henry, L. , & Müller, K. (2020). dplyr: A grammar of data manipulation. Retrieved from https://CRAN.R‐project.org/package=dplyr

[ece38409-bib-0128] Wickham, H. , Averick, M. , Bryan, J. , Chang, W. , McGowan, L. , François, R. , Grolemund, G. , Hayes, A. , Henry, L. , Hester, J. , Kuhn, M. , Pedersen, T. , Miller, E. , Bache, S. , Müller, K. , Ooms, J. , Robinson, D. , Seidel, D. , Spinu, V. , … Yutani, H. (2019). Welcome to the tidyverse. Journal of Open Source Software, 4(43), 1686. 10.21105/joss.01686

[ece38409-bib-0129] Wood, S. (2020). mgcv: Mixed GAM computation vehicle with automatic smoothness estimation. Retrieved from https://CRAN.R‐project.org/package=mgcv

[ece38409-bib-0130] Woodbridge, J. , Fyfe, R. , Smith, D. , Pelling, R. , Vareilles, A. , Batchelor, R. , Bevan, A. , & Davies, A. L. (2020). What drives biodiversity patterns? Using long‐term multidisciplinary data to discern centennial‐scale change. Journal of Ecology, 109, 1396–1410. 10.1111/1365-2745.13565

[ece38409-bib-0131] Xu, C. , Huang, Z. Y. X. , Chi, T. , Chen, B. J. W. , Zhang, M. , & Liu, M. (2014). Can local landscape attributes explain species richness patterns at macroecological scales?: Can landscape attributes explain richness patterns? Global Ecology and Biogeography, 23, 436–445. 10.1111/geb.12108

[ece38409-bib-0132] Yapp, R. (1922). The concept of habitat. Journal of Ecology, 10, 1–17. 10.2307/2255427

[ece38409-bib-0133] Zhang, Y. , Qian, L. , Spalink, D. , Sun, L. , Chen, J. , & Sun, H. (2021). Spatial phylogenetics of two topographic extremes of the Hengduan Mountains in southwestern China and its implications for biodiversity conservation. Plant Diversity, 43(3), 181–191. 10.1016/j.pld.2020.09.001 34195502 PMC8233532

[ece38409-bib-0134] Zolina, O. (2012). Change in intense precipitation in Europe. In Z. W. Kundzewicz (Ed.), Changes in flood risk in Europe. Special Publication 10, (pp. 97–119). IAHS Press.

[ece38409-bib-0135] Zurell, D. , Elith, J. , & Schröder, B. (2012). Predicting to new environments: Tools for visualizing model behaviour and impacts on mapped distributions. Diversity and Distributions, 18, 628–634. 10.1111/j.1472-4642.2012.00887.x

