# Supplementary figures and images for: Diversity of European habitat types is correlated with geography more than climate and human pressure
Source: Ecol Evol. 2021 Dec 7;11(24):18111–24. doi: 10.1002/ece3.8409 (PMC8717275; doi:10.1002/ece3.8409)

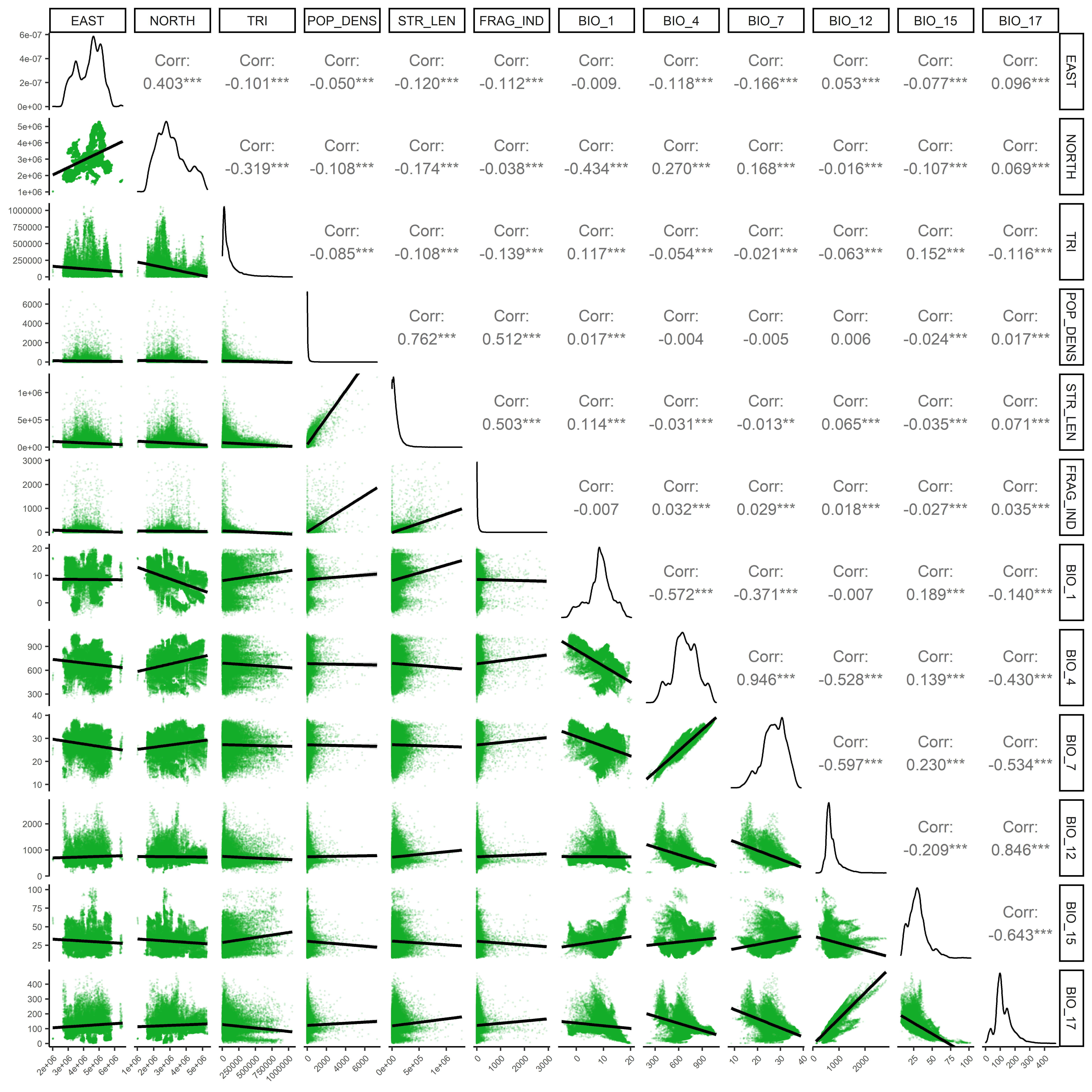

Supplement: Supplementary file 1 — Figure S1 [file ECE3-11-18111-s005.tiff]

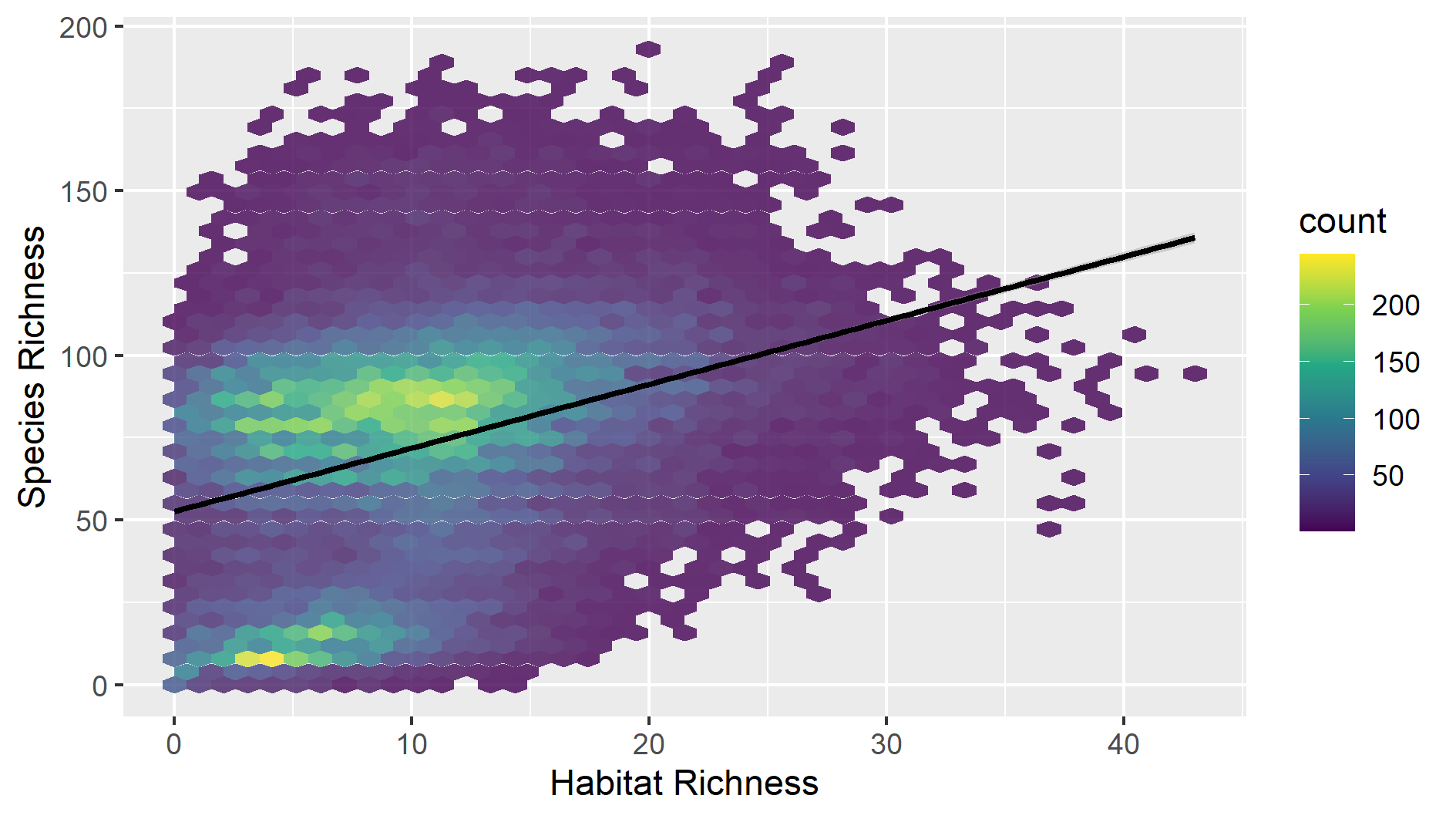

Supplement: Supplementary file 2 — Figure S2 [file ECE3-11-18111-s003.tiff]

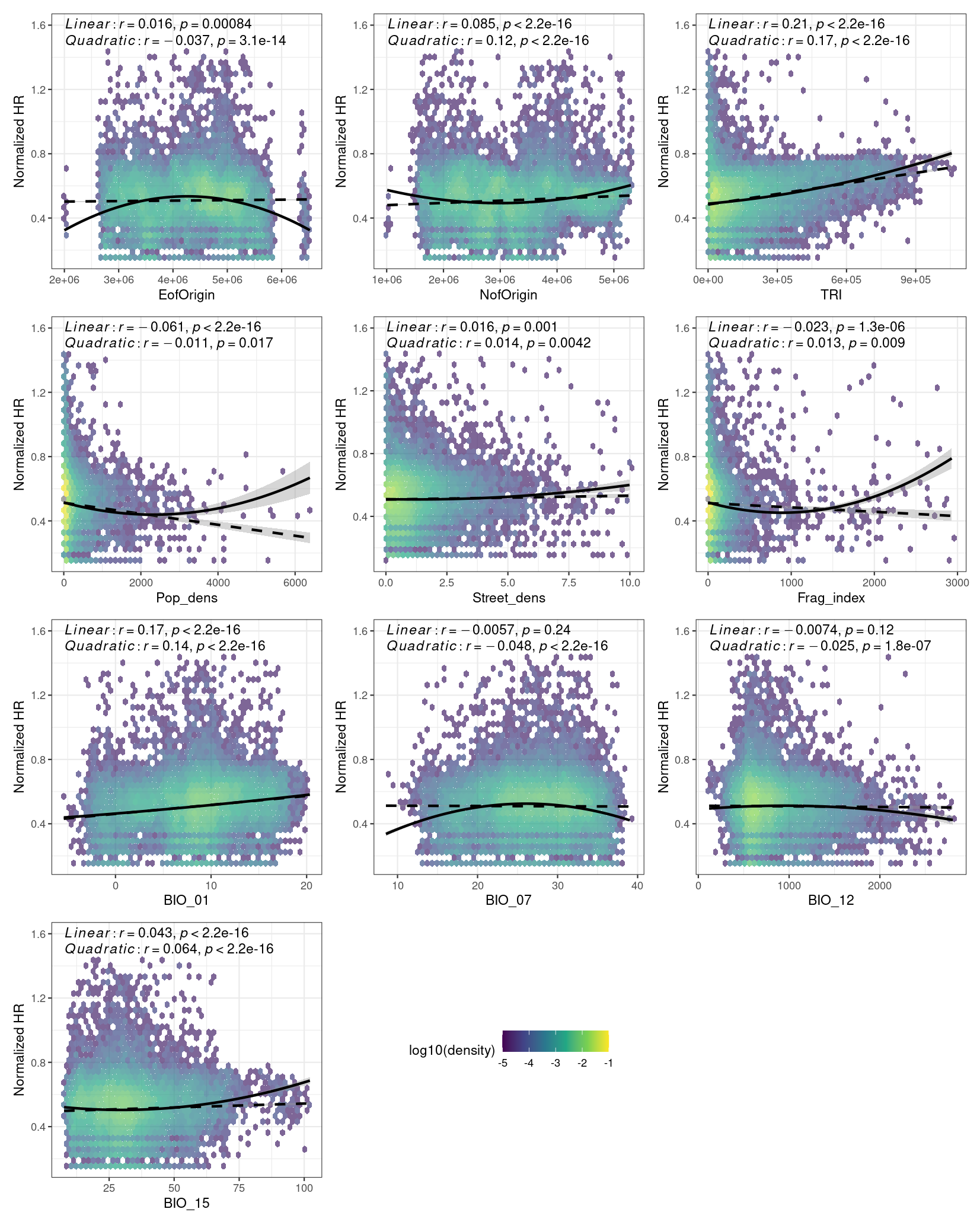

Supplement: Supplementary file 3 — Figure S3 [file ECE3-11-18111-s004.tiff]

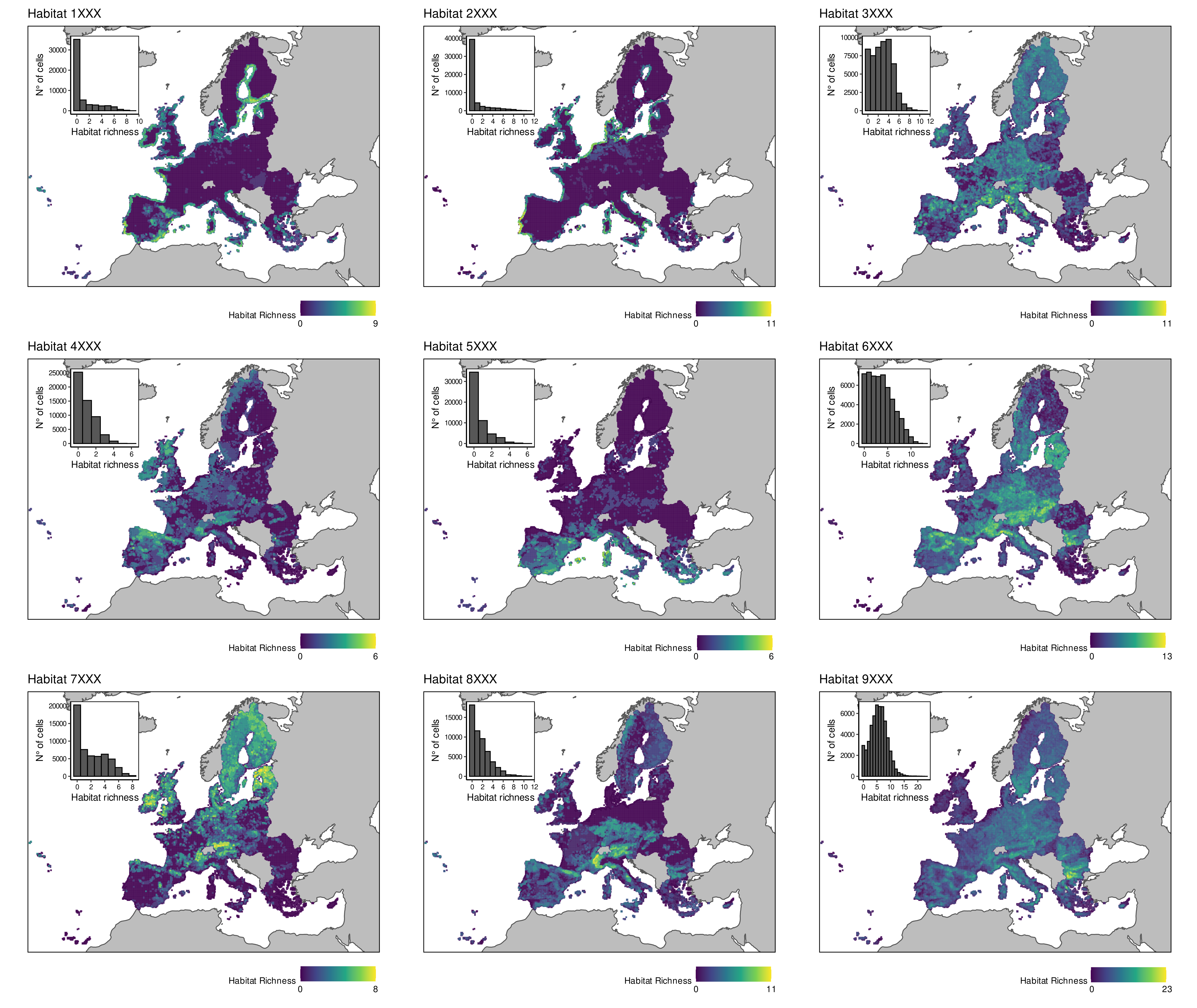

Supplement: Supplementary file 4 — Figure S4 [file ECE3-11-18111-s001.png]
