# Supplementary material for: Diversity of European habitat types is correlated with geography more than climate and human pressure
Source: Ecol Evol. 2021 Dec 7;11(24):18111–24. doi: 10.1002/ece3.8409 (PMC8717275; doi:10.1002/ece3.8409)
Supplement: Supplementary file 5 — Supplementary figure captions [file ECE3-11-18111-s002.docx]

Dear AE,

to be on the safe side, we attach the captions of the 4 supplementary figures because it does not seem to be possible to check them.

Kind Regards

**Supplementary material**

**Figure S1.** Correlation plots showing the correlation coefficients between all the explanatory variables.

**Figure S2.** Logarithmic-scale correlation (log-log) between Habitat Richness and the reported richness of the Annex species of the Birds and Habitats Directives.

**Figure S3.** Bivariate relationships between candidate explanatory variables and the response variable (Habitat Richness after normalization). Pearson'r correlation between first- and second-order polynomial explanatory variable and response variable, along with *p*-values, are shown in the panels.

**Figure S4.** Spatial distribution of habitat richness splitted into the 9 macro-categories. Histograms of habitat richness within 10 km x 10 km cells. Greece was not included in the analysis as explained in material and methods.
